# Supplementary material for: A biomimetic redox flow battery based on flavin mononucleotide
Source: Nat Commun. 2016 Oct 21;7:13230. doi: 10.1038/ncomms13230 (PMC5078740; doi:10.1038/ncomms13230)
Supplement: Supplementary Information — Supplementary Figures 1-10, Supplementary Tables 1-2 and Supplementary References [file ncomms13230-s1.pdf]

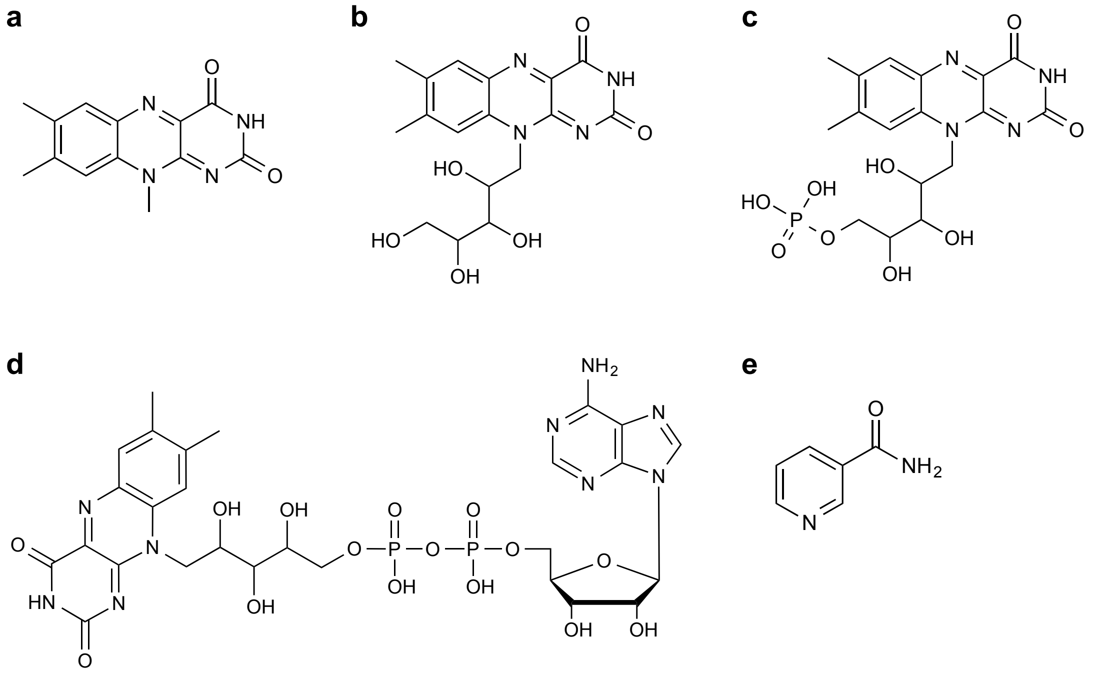

Supplementary Figure 1 | Flavins and nicotinamide . The molecular structures of (**a**) lumiflavin, (**b**) riboflavin, (**c**) flavin mononucleotide (FMN, riboflavin-5'-monophosphate), (**d**) flavin adenine dinucleotide (FAD), and (**e**) nicotinamide (pyridine-3-carboxylic acid amide).

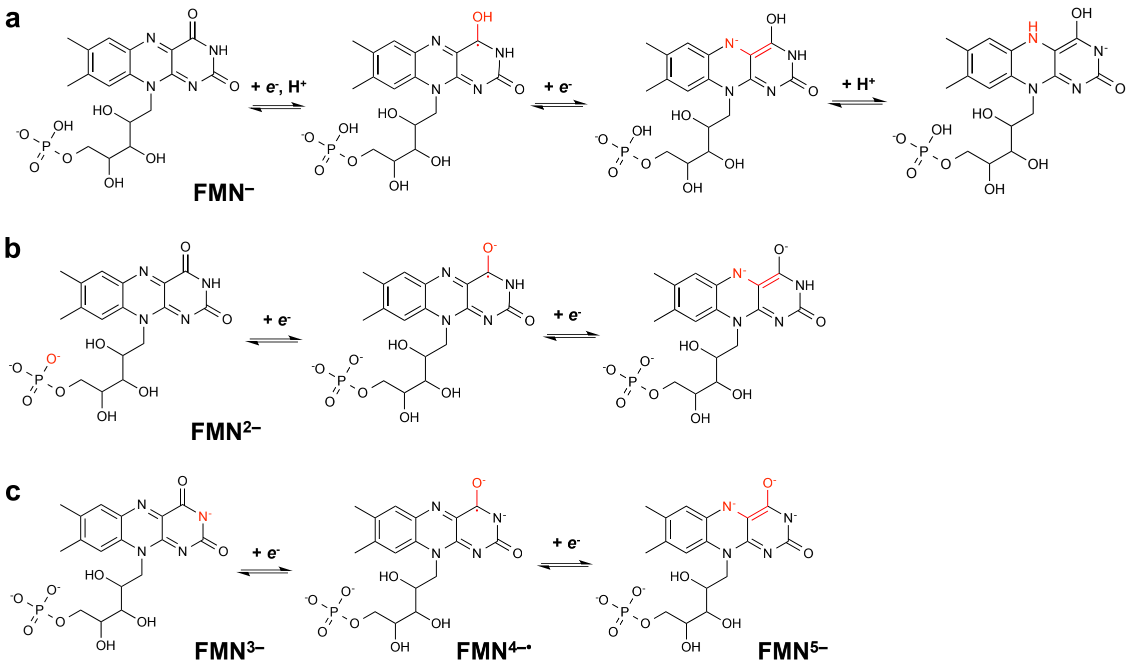

Supplementary Figure 2 | Redox reactions of FMN-Na. Proposed redox mechanisms of FMN-Na with different protonation states of the phosphate group and 3-position nitrogen. (**a**) pH = 5.5, (**b**) pH = 8.6, 10.0, and (**c**) pH = 13.0.

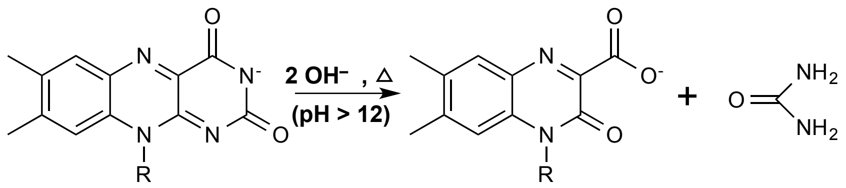

Supplementary Figure 3 | Alkaline hydrolysis of riboflavin.

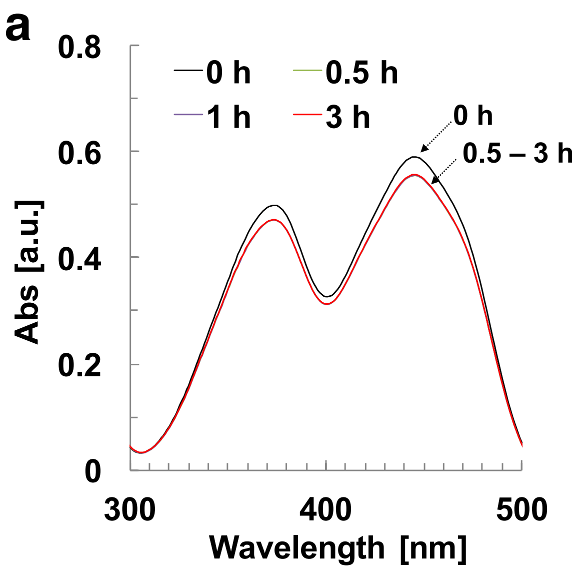

Supplementary Figure 4 | Dimerization effect on optical characteristics. UV-VIS spectra of (**a**) 50 μM FMN-Na (pH 5.5) and (**b**) 50 μM FMN-Na in pH 9.1 buffer (pH 8.6) aqueous solutions 0–6 h after electrolyte preparation.

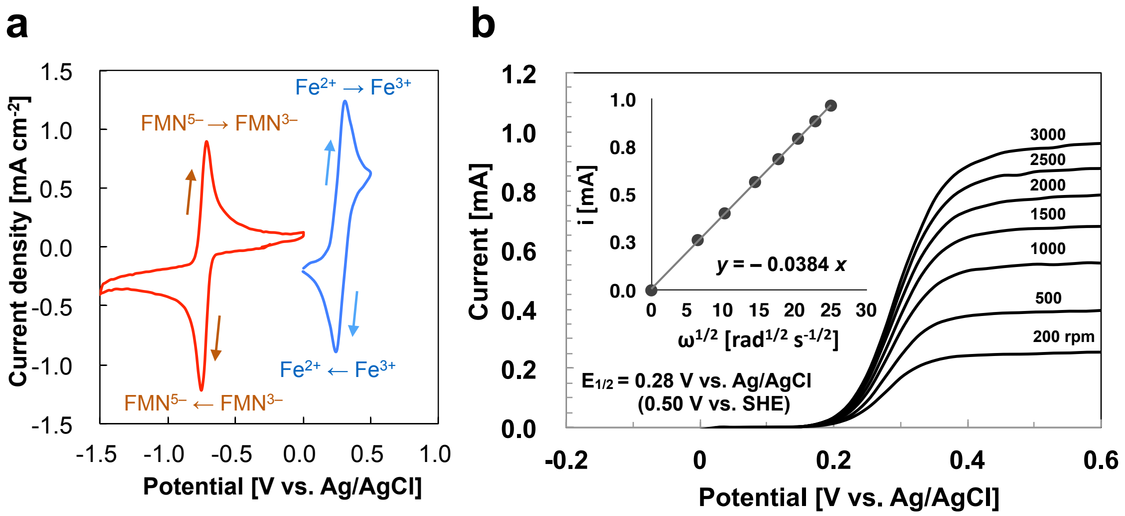

Supplementary Figure 5 | Electrochemistry of an electrolyte. (**a**) Cyclic voltammograms of 20 mM $K_4[Fe(CN)_6]$ and 1 M KOH aqueous positive electrolyte and 10 mM FMN-N and 1 M KOH aqueous negative electrolyte. (**b**) RDE measurements of 20 mM $K_4[Fe(CN)_6]$ and 1 M KOH aqueous positive electrolyte. The inset shows the limiting current (i) vs. the square root of the rotation velocity (Levich-plot).

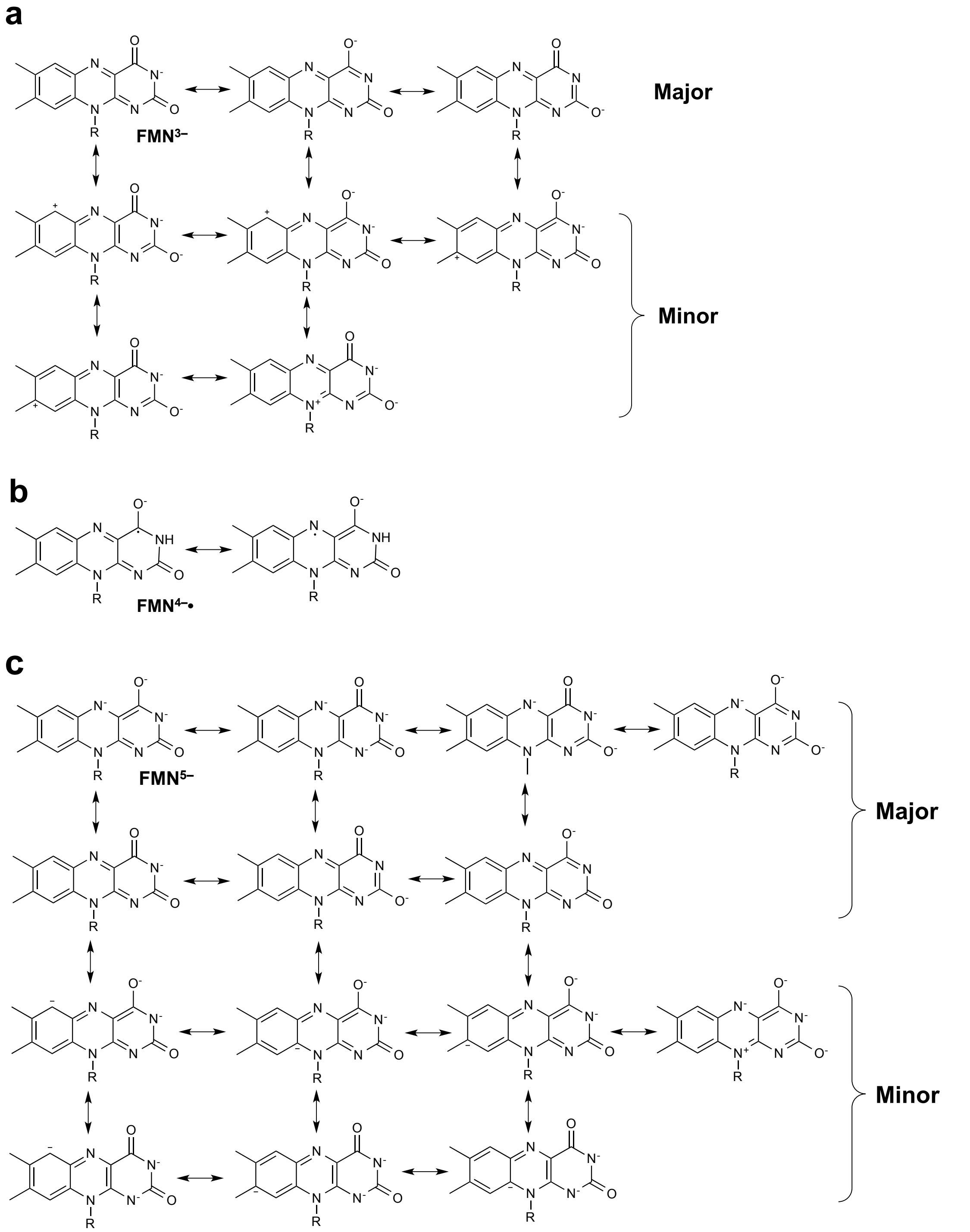

Supplementary Figure 6 | Possible resonance structures of FMN-Na. (**a**) FMN^{3-}, (**b**) $FMN^{4-\bullet}$, and (**c**) FMN^{5-}.

a

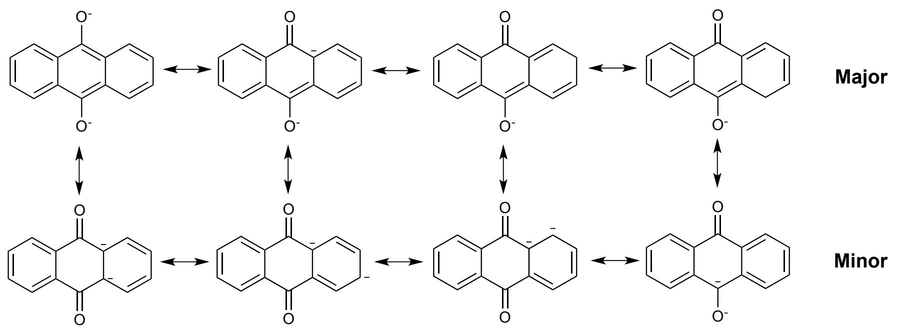

b

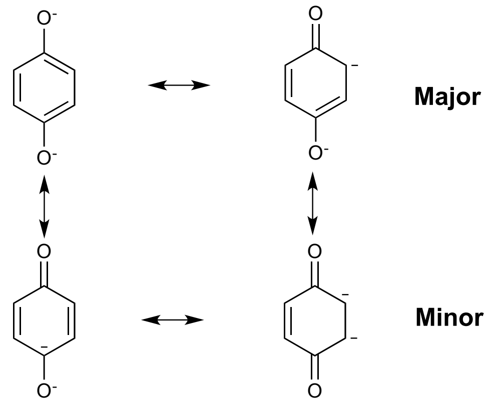

Supplementary Figure 7 | Possible resonance structures at two-electron reduced state. (**a**) Anthraquinone and (**b**) *p*-benzoquinone.

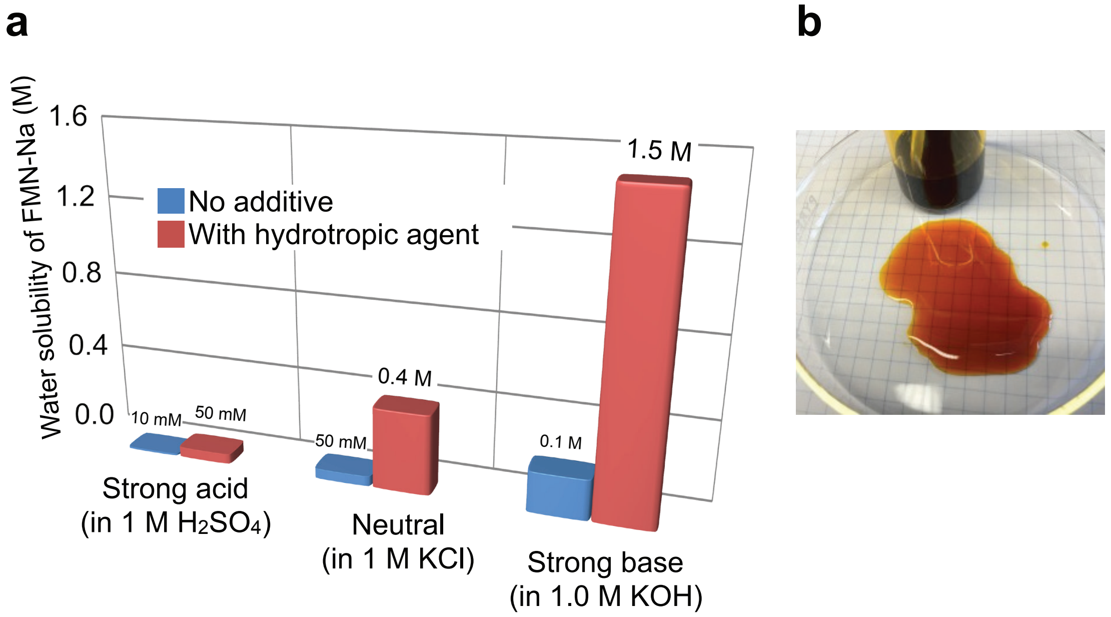

Supplementary Figure 8 | The effect of nicotinamide on water solubility of FMN-Na. (**a**) Water solubilities of FMN-Na in 1 M H_2SO_4, KCl, and KOH with and without 3 M nicotinamide (NA) at 288 K. (**b**) The picture of an aqueous electrolyte consisting of 1.5 M FMN-Na, 3.0 M NA, and 1.0 M KOH.

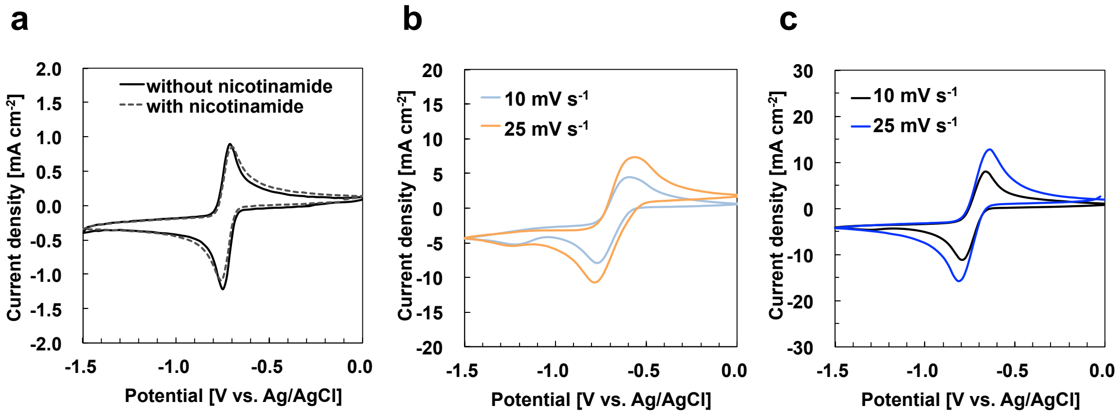

Supplementary Figure 9 | Electrochemistry of FMN-Na electrolytes with and without nicotinamide (NA). (**a**) Cyclic voltammograms (CVs) of 10 mM FMN-Na and 1 M KOH aqueous electrolytes with and without 10 mM NA at a sweep rate of 10 mV s^{-1}. (**b,c**) CVs of 0.24 M FMN-Na, 1 M NA, and 1 M KOH aqueous electrolyte at a sweep rate of 10 and 25 mV s^{-1}. CVs in (**a**) and (**c**) were measured immediately after electrolyte preparation. CV in (**b**) was measured 100 h after electrolyte preparation.

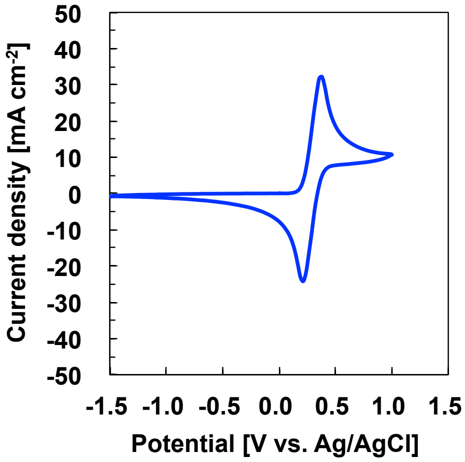

Supplementary Figure 10 | Electrochemistry of a positive electrolyte after 200 cycles. Cyclic voltammogram (CV) of 0.4 M $K_4[Fe(CN)_6]$ and 1 M KOH aqueous electrolyte at a sweep rate of 25 mV s^{-1}.

Supplementary Table 1 | Kinetic parameters of redox couples in aqueous solutions.

Redox couple	Kinetic rate constant k [cm s^{-1}]	Concentration of redox species [mM]	Supporting electrolyte	Electrode	Ref.
V^{2+}/V^{3+}	2.2×10^{-5}@ 298 K	50	1 M H_2SO_4	PFC[1)]	1
VO_2^{+}/VO^{2+}	6.8×10^{-5}@ 298 K	50	0.5 M $HClO_4$	GC[2)]	1
Fe^{2+}/Fe^{3+}	2.2×10^{-5}@ 298 K	40	0.5 M $HClO_4$	Au	2
Fe^{2+}/Fe^{3+}	5×10^{-3}@ 293 K	1	1 M $HClO_4$	Pt	3
$[Fe(CN)_6]^{4-}$ /$[Fe(CN)_6]^{3-}$	9×10^{-2}@ 293 K	1	1 M KCl	Pt	3
$AQDS/AQDS^{2-}$	$7.2(5)\times10^{-3}$	1	1 M H_2SO_4	GC	4
FMN^{3-}/FMN^{5-}	$(5.8\pm\ 0.6)\times10^{-3}$ @ 298 K	10	1 M KOH	GC	This work

1) PFC: Plastic formed carbon, 2) GC: Glassy carbon

AQDS: 9,10-anthraquinone-2,7-disulphonic acid

Supplementary Table 2 | Comparison of aqueous redox flow batteries based on organic active materials.

Active material (concentration)		Energy Density[1)] [Wh L^{-1}]	Max. power density [W cm^{-2}]	Capacity retention (100 cycles) [%]	Ref.
Positive	Negative				
TEMPO-polymer (N/A)	Viologen-polymer (N/A)	~3.6[2)]	N/A	~ 80	5
TEMPOL (0.5 M)	Methyl-viologen (0.5 M)	~ 4.7[3)]	N/A	~85	6
Bromide/bromine (3 M HBr/0.5 M Br_2)	AQDS (1 M)	~ 16[4)]	1.0	~ 100	4,7,8
$K_4[Fe(CN)_6]$ (0.4 M)	DHAQ (0.5 M)	N/A[5)]	0.40	~ 90	9
$K_4[Fe(CN)_6]$ (0.4 M)	FMN-Na (0.24 M)	4.8	0.16	~99	This work

1) Measured energy density was calculated from the capacity (Ah L^{-1}) and average discharge voltage. Volume is based on the total volume of positive and negative electrolytes.

2) Capacity: (10 Ah L^{-1} × 10 mL)/(10 mL + 15 mL) = 4 Ah L^{-1}; average discharge voltage: *ca.* 0.9 V.

3) Capacity: (10.5 Ah L^{-1})/2 = 5.3 Ah L^{-1}; average discharge voltage: *ca.* 0.9 V.

4) 40 °C. Capacity: 26.8 Ah L^{-1}; average discharge voltage: *ca.* 0.6 V.

5) 45 °C. The only normalized capacity was shown in ref 9.

TEMPOL: 4-hydroxy-2,2,6,6-tetramethyl-1-piperidinyloxyl,

AQDS: 9,10-anthraquinone-2,7-disulphonic acid ,

DHAQ: 2,6-dihydroxyanthraquinone.

Supplementary References

1 Yamamura, T., Watanabe, N., Yano, T. & Shiokawa, Y. Electron-transfer kinetics of Np^{3+}/Np^{4+}, NpO^{2+}/NpO_2^{2+}, V^{2+}/V^{3+}, and VO_2^{+}/VO^{2+} at carbon electrodes. *J. Electrochem. Soc.* **152**, A830-A836 (2005).

2 Weber, A. Z. *et al.* Redox flow batteries: a review. *J. Appl. Electrochem.* **41**, 1137-1164 (2011).

3 Randles, J. E. B. & Somerton, K. W. Kinetics of rapid electrode reactions. Part 3. Electron exchange reactions. *Trans. Faraday Soc.* **48**, 937-950 (1952).

4 Huskinson, B. *et al.* A metal-free organic-inorganic aqueous flow battery. *Nature* **505**, 195-198 (2014).

5 Janoschka, T. *et al.* An aqueous, polymer-based redox-flow battery using non-corrosive, safe, and low-cost materials. *Nature* **527**, 78-81 (2015).

6 Liu, T. B., Wei, X. L., Nie, Z. M., Sprenkle, V. & Wang, W. A total organic aqueous redox flow battery employing a low cost and sustainable methyl viologen anolyte and 4-HO-TEMPO catholyte. *Adv. Energy Mater.* **6**, 1501449 (2016).

7 Chen, Q., Gerhardt, M. R., Hartle, L. & Aziz, M. J. A quinone-bromide flow battery with 1 W/cm^2 power density. *J. Electrochem. Soc.* **163**, A5010-A5013 (2016).

8 Huskinson, B., Marshak, M. P., Gerhardt, M. R. & Aziz, M. J. Cycling of a quinone-bromide flow battery for large-scale electrochemical energy storage. *ECS Trans.* **61**, 27-30 (2014).

9 Lin, K. X. *et al.* Alkaline quinone flow battery. *Science* **349**, 1529-1532 (2015).
